# Macrophage Polarization in Virus-Host Interactions

**DOI:** 10.4172/2155-9899.1000311

**Published:** 2015-03-27

**Authors:** Yongming Sang, Laura C Miller, Frank Blecha

**Affiliations:** 1 Department of Anatomy and Physiology, College of Veterinary Medicine, Kansas State University, Manhattan, KS 66506, USA; 2 Virus and Prion Diseases of Livestock Research Unit, National Animal Disease Center, USDA-ARS, 1920 Dayton Ave, Ames, IA 50010, USA

**Keywords:** Macrophage polarization, Viral infection, Antiviral state, Interferon, Virus-host interaction

## Abstract

Macrophage involvement in viral infections and antiviral states is common. However, this involvement has not been well-studied in the paradigm of macrophage polarization, which typically has been categorized by the dichotomy of classical (M1) and alternative (M2) statuses. Recent studies have revealed the complexity of macrophage polarization in response to various cellular mediators and exogenous stimuli by adopting a multipolar view to revisit the differential process of macrophages, especially those re-polarized during viral infections. Here, through examination of viral infections targeting macrophages/monocytic cells, we focus on the direct involvement of macrophage polarization during viral infections. Type I and type III interferons (IFNs) are critical in regulation of viral pathogenesis and host antiviral infection; thus, we propose to incorporate IFN-mediated antiviral states into the framework of macrophage polarization. This view is supported by the multifunctional properties of type I IFNs, which potentially elicit and regulate both M1- and M2-polarization in addition to inducing the antiviral state, and by the discoveries of viral mechanisms to adapt and modulate macrophage polarization. Indeed, several recent studies have demonstrated effective prevention of viral diseases through manipulation of macrophage immune statuses.

## Macrophage Polarization is Associated with Viral Infections and Antiviral States

### Origin and retention of macrophages

**Figure 1:**
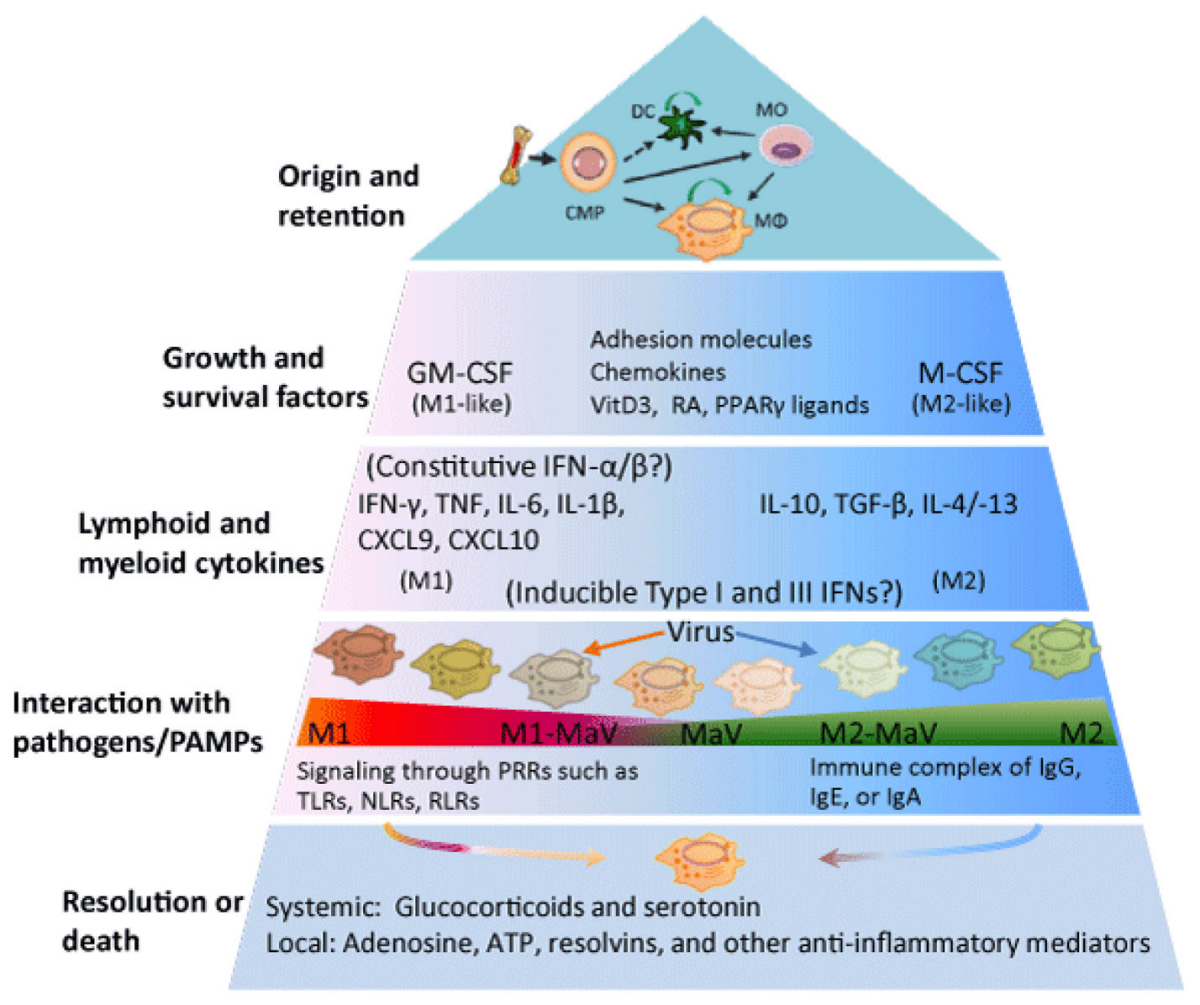
Incorporation of the antiviral state into a multilayer scheme of macrophage polarization. The top panel illustrates recent findings about the origin and self-renewal property of tissue macrophages. In contrast to the dichotomy system for addressing macrophage polarization that classifies macrophages either as classic (M1) or alternative (M2) activation statuses, a multipolar view has been proposed to revise macrophage polarization based on a much broader functional repertoire for macrophages mediated by various mediators/stimuli grouped in different layers [1,6]. Antiviral state (MaV), which is a cell-autonomous status to restrict virus infection and replication in response to viral infection or IFN stimulation, has not been well integrated into the paradigm of macrophage activation. In line with our previous work to study MaV in the framework of macrophage activation [70], here we elaborate the potential diversity of MaV states corresponding to the multifunctional properties of type I and type III IFNs as shown in Figure 2. CMP: Common Myeloid Progenitor; CXCL: Chemokine C-X-C Motif Ligand; DC: Dendritic Cells; (G)M-CSF: (Granulocyte-) Macrophage Colony-Stimulating Factor; MO: Monocyte; MΦ: Macrophage; NLR: NOD-Like Receptors; PPARγ: Peroxisome Proliferator-Activated Receptor gamma; PRR: Pathogen Recognition Receptor; RA: Retinoic Acid; RLR: RIG-Like Receptor; TLR: Toll-Like Receptor; VitD3: Vitamin D3. Modified from Martinez and Gordon [6].

Macrophages (MΦs), together with blood monocytes (MOs) and dendritic cells (DCs), comprise a mononuclear cell lineage that originates from common myeloid progenitors. During normal development and various pathophysiological processes, tissue-resident MΦs are largely differentiated from circulating MOs and, self-renew locally from MΦs of embryonic origin (Figure 1) [1–6]. Macrophages are distributed widely in the body, where they adhere to various mucosal surfaces or mingle with other cell types of different tissues. Tissue-resident macrophages specific to certain anatomic locations include blood monocytes, peritoneal macrophages, pulmonary macrophages, Kupffer cells in the liver, and microglia in the brain [2–5]. Since the original description of “phagocytes” by Metchnikoff [7,8] Macrophages can be further diversified according to different micro-anatomical locations; for example, pulmonary macrophages are divided into three subgroups with respect to their contacting microenvironments in the lung: alveolar macrophages, interstitial macrophages, and intravascular macrophages [2,9,10]. Accordingly, MΦs in different tissues show dramatic phenotypic specialization corresponding to their functional diversity [2,11], numerous studies have addressed the broad roles of MΦs in normal development and, in particular, in pathophysiological processes involved in inflammation, pathogen clearance, wound healing, tissue regeneration, angiogenesis, tumor/cancer progression, and the development of obesity [12–18]. Immunologically, MΦs belong to innate immune cells and conserve their immune surveillance, inflammatory regulation and phagocytic activity during pathogenic infection throughout the animal kingdom [1,2,11]. However, the evolution of adaptive immunity in higher vertebrates endows MΦs with functions associated with both T and B cell responses [1–3,19]. In this context, MΦs, along with the professional antigen presentation cells (APCs) DCs, serve as a major group of non-professional APCs bridging and regulating adaptive immunity. One characteristic of MΦs that profoundly contributes to their adaptability to the surrounding anatomic microenvironment is their versatile plasticity. The functional plasticity of MΦs arises from their capacity to respond to cellular mediators and exogenous stimuli. During pathogenic infection, for example, they demonstrate a wide variety of activation (polarization) statuses involved in the progression and outcomes of various pathogen-host interactions [2,3,4,5,6,11].

### A multipolar view of macrophage polarization and associations with viral infections

Studies of MΦ activation statuses, as represented by the classical (M1) and alternative (M2) activation statuses, have been associated primarily with bacterial and parasitic infections, respectively [1,3,6]. The M1 and M2 statuses represent cell activation statuses polarized by cytokines, initially determined using interferon (IFN)-γ and interleukin (IL)-4/IL-13 that are typically secreted by Th1 or Th2 cells, respectively. Consequentially, MΦ activation statuses identified later have been termed M1- or M2-like statuses. The M1-like status includes those polarized by single or a combination of Th1 cytokines and pro-inflammatory mediators including granulocyte-macrophage colony-stimulating factor (GM-CSF), tumor necrosis factor (TNF)-α, IL-6, IL-1β, IL-12, and various pathogen-associated molecular patterns (PAMPs). In contrast, M2-like statuses cover those polarized by macrophage colony-stimulating factor (M-CSF), immune complex (known as M2b), IL-10 (known as M2c), as well as glucocorticoid and serotonin. A more recent proposal hypothesized that, rather than a distinctly bipolar M1/M2 paradigm, a continuum or spectrum of macrophage activation states exists with the many mediator/stimuli cohorts in resident tissues [1–6,11–13,20]. This complexity of MΦ polarization calls for revisiting the M1/M2 dichotomy initially characterized using only a few selected ligands. To this end, Martinez and Gordon provided a multipolar view of MΦ polarization in an immunological context [1,6]. They proposed that according to their role in MΦ differentiation and immune responses, mediators/stimuli for MΦ polarization could be organized at four levels: growth and survival factors, lymphoid and myeloid cytokines, interaction with pathogens and PAMPs, and signaling molecules leading to resolution (Figure 1).

This model of organizing a variety of mediators for MΦ polarization into a developmental and immunological context is appropriate. However, we propose to integrate viral infection and the IFN-stimulated antiviral state into this paradigm (Figure 1) [1,6]. Considering the diverse pathogenic processes of viral infections caused by different viral species and strains of the same species, simply ascribing MΦs polarized by viral infection or IFN treatment to M1-like statuses is too inclusive to reflect the heterogeneity of viral pathogenesis and the functional diversity of type I and type III IFNs. Instead, it is more appropriate to inspect what underlies viral infections in a case-dependent and dynamic way [14–16,21–26]. In addition, because of the multifunctional properties of type I and type III IFNs, the antiviral state induced by type I and type III IFNs may result in different polarizing potencies in MΦs [27–31]. For example, in addition to boosting antiviral responses through induction of IFN-stimulated genes (IFNs) and pro-inflammatory cytokines (i.e., for M1-like polarization), IFN-α/-β (type I IFNs) and IFN-λs (type III IFNs) also potently stimulate the production of IL-10 and other immunosuppressive responses (M2-like) during persistent viral infection (See Section 3 for detail) [27–31].

Because of their diverse distribution in the body and the critical role of monocytic cells in immune regulation, multiple viruses have evolved to infect and replicate in both differentiated MΦs and their precursor MOs (Table 1) [32–66]. Either by direct infection or through sensing infections in other cells, MΦs are inevitably skewed into different functional phenotypes, thereby interacting with both viral pathogenesis and host antimicrobial responses. Indeed, most monocytotropic viral infection, such as those caused by HIV, RSV, SARS, and IAV (Table 1), may affect MΦ polarization, and in turn oblige the host with the outcome of immunosuppression and/or immunopathology; these processes are generally associated with viral persistence and co-infections by pathogens of other phyla [67–69]. In this regard, through studying monocytotropic viral infections, we and others have recently proposed integrating antiviral states into the framework of MΦ polarization for managing antiviral responses [6,10,14,16,70,71]. This is imperative not only for antiviral regulation per se, but also for studies of immune regulation and general antimicrobial responses underlying MΦ biology [1,2,6,10]. In this review, we examine cell polarization related to direct viral infection and IFN-stimulated antiviral states in MΦs and related monocytic cells. Here, we will discuss this topic primarily by using examples of respiratory viral infections in humans and animals (Table 1).

## Macrophage Polarization Interacts with Viral Infections

### Macrophage polarization response to viral infections

Until recently, MΦ polarization or activation statuses have been studied exclusive of viral infection. Similarly, studies of antiviral states in macrophages have involved little attention on typical activation statuses, even though typical cytokines for macrophage polarization such as IFN-γ, IL-4, and IL-10 are rigorously regulated during monocytotropic viral infections. The interaction of viral infections with MΦ polarization has been directly demonstrated in HIV and RSV infections, and associated with infections caused by human herpes viruses, influenza, SARS, and other viruses (Table 1).

In human monocyte-derived macrophages (MDMs), HIV-1 infection skewed cells toward a M1-like status, which correlated with downregulation of M2-status markers (CD163, CD206, CCL18, and IL-10) and increased secretion of M1-associated chemokines including CCL3, CCL4, and CCL5 (ligands of CC-chemokine receptor 5 (CCR5), the main HIV-1 entry receptor). Unlike the typical M1-status stimulated by LPS (or IFN-γ), these HIV-1 polarized M1-like macrophages were hyperresponsive to microbial stimuli via toll-like receptors (TLRs) but independent of the production of pro-inflammatory cytokines including IL-1β and IL-6. Thus, these HIV-1 polarized M1-like macrophages probably had less antimicrobial activity and likely were more “inflamed” than typical M1 macrophages. In fact, either typical M1- or M2-statuses activated using IFN-γ (plus TNF-α) or IL-4 in MDMs were shown to be less supportive of CCR5-dependent (R5) HIV-1 replication than control MDMs. Further studies reported that the IFN-γ-mediated M1 status restricted HIV-1 replication at a preintegration step via downregulation of primary CD4 receptors and CCL chemokines (CCL3, CCL4, and CCL5), and M2a polarization inhibited viral replication at a post-integration level. Therefore, HIV-1 infection likely acts on MΦ polarization to change the cell permissiveness and alter the outcome of the infection [14,72–76].

Similarly, MΦ polarization is likely involved in RSV infection [77,78]. When virus-induced bronchiolitis, in association with a mixed “Th1” and “Th2” cytokine storm, occurred [77,78], non-selective depletion of lung macrophages abolished the increase of inflammatory cytokines at 1 day post-infection (dpi) and enhanced viral load in the lung at 4 dpi. This suggests an important role of lung MΦs and their polarization (probably M1-like) in control of viral replication [34,35]. In mice deficient in the IL-4 receptor, thus, blocking M2a polarization in MΦs, RSV infection exacerbated lung inflammation and injury, indicating that balanced M2 differentiation is essential for controlling RSV-induced immunopathology at the later stages of the disease [77,78]. Therefore, the involvement of MΦ polarization in RSV infection and its contribution to either viral pathogenesis or host antiviral response changes as the viral disease progresses. Herbein and Varin (2010) have proposed a model mostly based on retroviral infections, in which macrophages are dynamically polarized during the course of a disease, with an M1-phenotype dominating during the early phase and an M2a-profile emerging during the chronic phase of the disease, eventually leading to macrophage deactivation depending if the virus is under control or if the host becomes tolerant [14].

### Viral infections affect the progression of macrophage polarization

The progressive pattern of MΦ polarization described above should prevent most viral attacks that animals experience. However, most pathogenic attempts likely have been eliminated before notable shifts of MΦ polarization. In this regard, most notorious viruses have evolved mechanisms to eliminate MΦs, compromise MΦ functions, and divert the proper progression of MΦ polarization. A typical strategy for most highly pathogenic viruses to cause severe pathology is to incite M1-associated inflammation, which not only promotes viral spreading via increased lymphocyte flux (including the infected monocytic cells), but also causes massive cell death of MΦs through direct infection. This has been demonstrated in diseases such as SARS [44–46], pandemic influenza [36–38], ASFV [55,56] and high-pathogenic PRRSV [64,65,71] (Table 1). As shown in these highly-pathogenic viruses, infections cause as much as 50% MΦ depletion through apoptosis and necrosis, which are mostly M1-like status cells with a higher antiviral/inflammatory activity but short lifespans [44–46,73,75]. In an in vitro test, infection by Ebola virus led to 60–70% death of infected monocytes/macrophages and 40% bystander death of T cells in human peripheral blood mononuclear cells (PBMCs) at 4 dpi [79]. Virus-mediated massive cell death led to a series of pathological consequences associated with MΦ polarization even if the hosts survived the acute infections: (1) diminishing the first-line antiviral defense performed by these M1-like MΦs, thus facilitating acute viral replication as shown in most artificial MΦ-depletion assays (Table 1); (2) attenuating secondary antiviral signaling (virus-infected MΦs represent a primary type I IFNs producer) and M1 mediators (inefficiently bridging Th1 cells to produce IFN-γ) to polarize the influx of monocytes in place of the depleted MΦs [44–46,73,75,80]; (3) causing tissue damage, thus inducing M2-like status of resident MΦs for wound healing before viral clearance [14,78] and (4) causing viruses to hijack the vulnerable M2-cells to form a systemic or persistent infection and retard homeostatic resolution (Figure 1, layers 3–5) [14]. In brief, these highly-pathogenic viruses subvert the MΦ polarization cascade that has been programmed to confront regular viral infections by inciting acute inflammation (cytokine storm) and cell death. The production of the pro-inflammatory “cytokine storm” may “burn” macrophages into an “over-inflamed” status rather than typical M1 or antiviral states [81,82]. Similar to the different antiviral phenotypes in HIV-1-mediated M1 with typical M1 status, these “over-inflamed” macrophages probably injure themselves and the host rather than exerting effective antimicrobial responses [19,72]. Unfortunately, related studies about the authentic phenotypes and lifespans of these “over-inflamed” macrophages, and how they differ from typical M1-status, are lacking. These “over-inflamed” macrophages, in part, may correspond to the Th17 response and mimics a novel identification of Th17 polarization induced during mycobacterial infection [19,72]. Suppression of the virus-induced cytokine storm through different signaling pathways could protect patients from lethal influenza infection even without diminishing viral replication [72,82]. Similar modulation to increase M2a differentiation blunted RSV-mediated lung pathology [16,77].

Macrophages at different activation statuses have corresponding functional phenotypes. M1-macrophages are characterized as proinflammatory, tissue destructive, anti-tumoral, antimicrobial, and immunogenic; in contrast, M2-macrophages are anti-inflammatory, tissue repairing, pro-tumoral, tolerogenic, and regulatory [2,3,6,11,12,13]. Viral infections in MΦs may alter functional phenotypes to some extent with or without full repolarization. Regarding the host, successful antiviral responses pertaining to infected or bystander MΦs might strengthen the cells toward M1 and antiviral states (M1-MaV), which enhance their capacity to inactivate the viruses and signal sequential immunity. Viruses often evolve mechanisms to enhance M2-prone responses. One strategy is to subvert or re-circuit the host cytokine network. Because one key feature of IL-10 is to induce M2-polarization and exert potent immunosuppressive effects [83–85], several viruses have been shown to upregulate the expression of IL-10 [86]. Examples include hepatitis C, FMDV, measles virus, and PRRSV during infection of monocytic cells, and HIV-1 during viremic persistence [83–86]. More autonomously, other viruses, including members of herpesviruses, alloherpesviruses and poxviruses, encode functional orthologues of IL-10, called viral IL-10s (vIL-10s) [87]. Due to the pleiotropic function of IL-10-mediated signaling in immunosuppression and cell differentiation (both T regulatory cells and M2c cells), viruses evolving these mechanisms are likely capable of masking host antiviral responses and causing persistent and systemic infections [83–87]. Porcine macrophages infected by classical swine fever virus (CSFV) showed an increase in the M2-marker arginase-1 (ARG-1) but a decrease in nitric oxide production, indicating a M2-prone polarization [55]. In this respect, we and others have shown that PRRSV infection in macrophages stimulated IL-10 production, and cells of all M2 statuses. In particular, IL-10-mediated M2c status were significantly more permissive to PRRSV infection [10,70,71]. In summary, the frequent occurrence of monocytotropism in viral infections and related viral mechanisms in regulating macrophage polarization imply an essential role of the proper progression of macrophage polarization in the virus-host interaction and disease outcomes.

## Antiviral Interferons (Type I and Type III IFNs) Potentiate and Regulate Macrophage Polarization

### Critical role of constitutive weak IFN-α/β-signaling

Solely ascribing macrophages polarized by viral infection as a M1-like status is counter-indicated by the molecular and functional complexity of both type I and type III IFNs, the cytokines primarily known for eliciting an antiviral state [28–31]. However, recent studies of the molecular and functional diversity of these antiviral IFNs have revisited their role in macrophage polarization. Although designated as “interferons”, type I and type III IFNs have much more molecular diversity than the type II IFN, IFN-γ. For example, most mammalian species have 17–60 and 2–4 functional genes within type I and type III IFN gene loci, respectively [31,88]. Relative to protein structural signatures, type III IFNs actually belong to the IL-10-cytokine family whose receptors consist of a common IL-10R2 receptor chain [31]. The three families of IFNs are perceived by distinct cognate receptors (IFNAR1/IFNAR2, IFNGR1/IFNGR2, and IFNλR1/IL-10R2 for type I, II and III IFNs, respectively), which in turn mediate cell signaling pathways that crosstalk and are virtually similar between those responsive to type I and type III IFNs [28–31]. The receptors for type I IFNs are present on most cell types, but those for type II and type III IFNs are mostly expressed on hematopoietic cells (NK, NKT, Th1, and CTL cells) and epithelial cells, respectively [28–31]. Receptors of all three families of IFNs are present in macrophages and their gene expression levels change only marginally (1–2 fold) with macrophage polarization (unpublished data) [70,71], indicating that macrophages may remain responsive to IFNs independent of polarization status [28–31].

The production of type I and type III IFNs previously was thought to be restricted to cells upon viral infection or related stimuli. However, recent studies have revealed low levels of constitutively produced IFN-α/β independent of viral challenge by mouse embryonic fibroblasts (MEFs) and mononuclear phagocytic cells in peripheral tissues [89–92]. Further studies have indicated that this cell-intrinsic IFN signaling is critical to cell transformation [87] and potentiates cell responses to IFN-γ, IL-6, and later-induced type I IFNs [93,94]. First, signaling by IFN-γ depends on a type I IFN receptor component, IFNAR1, which facilitates efficient assembly of IFN-γ-activated transcription factors. This cross talk is contingent on a constitutive subthreshold IFN-α/β signaling and the association between the two nonligand-binding receptor components, IFNAR1 and IFNGR2, in the caveolar membrane domains [93]. Second, constitutive subthreshold IFN-α/β signaling also contributes to efficient IL-6 signaling. In effect, IL-6-induced activation of transcription factors (i.e., signal transducer and activator of transcription (STAT)1 and STAT3) is markedly diminished in the absence of subthreshold IFN-α/β signaling [94]. In this case, the weak IFN-α/β stimulation promotes IFNAR1 phosphorylation at its cytoplasmic tyrosine residues, which provides docking sites for STAT1 and STAT3 to form homo- or heterodimers following IL-6 stimulation and induces interaction with gp130, a common signal transducer for the IL-6 family of cytokines [94]. Third, using IFN-α/β, it has been demonstrated that type I and type III IFNs have a positive self-regulatory loop; i.e., the early subthreshold IFNs potentiate robust IFN responses and induction of an antiviral state after viral infection [28–31]. Therefore, the constitutive weak and early IFN-α/β signaling may provide a foundation for strong cellular responses to antimicrobial polarization by IFN-γ (M1) [93], IL-6 (pro-inflammatory) [94], antiviral IFNs [26–29], and possibly other cytokines [29,93,94]. Thus, to fit in the multipolar model of macrophage polarization (Figure 1), constitutive subthreshold IFN-α/β signaling may be more hierarchical than adaptive IFN-γ and other inducible cytokines (including type I and type III IFNs produced later during viral infections) in M1 (or antimicrobial) polarization [6]. Indeed, it is compatible with the presence of pDCs (and potentially other cell types as described above) as autonomous IFN-α producers [95] and later sequential production of adaptive IFN-γ and other inducible cytokines (by adaptive activation of lymphoid and myeloid cells) in antiviral immune responses [28–30]. Based on the observations and discussion above, we propose that the constitutive subthreshold IFN-α/β signaling is critical to efficient induction of M1 and MaV states in macrophages [89–94]. The related unanswered questions are what mechanisms regulate the production of such constitutive subthreshold IFN-α/β in peripheral tissues (see Section 5); and whether type I and type III IFNs also affect M2-like statuses in macrophages.

### Potency of type I and type III IFN signaling to affect M1- and M2-statuses

**Figure 2:**
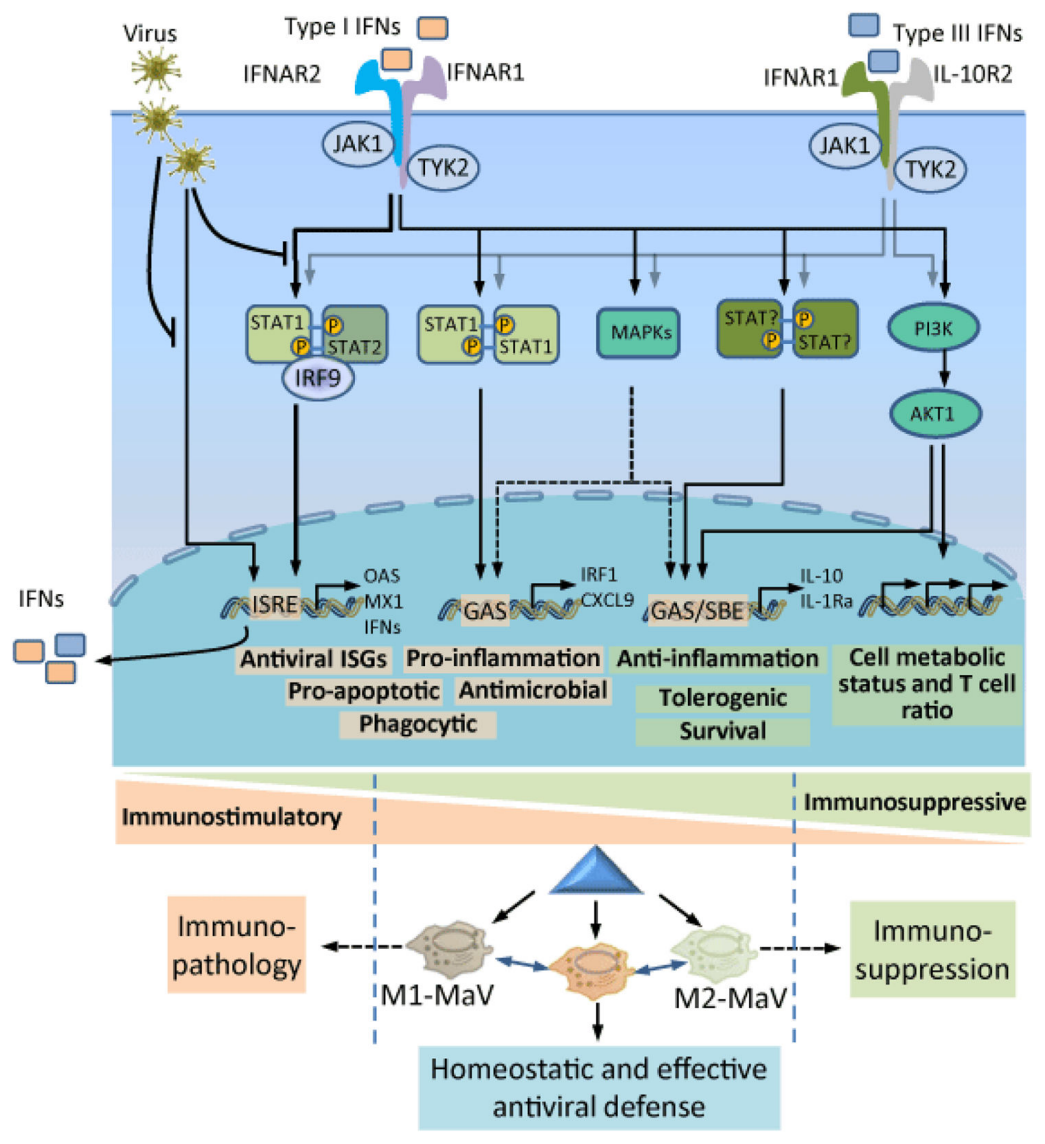
Ramification of IFN signaling pathways leading to immunostimulatory and immunosuppressive regulation of macrophage polarization. Viral infection of tissue-resident macrophages or nearby cells leads to production of type I and type III IFNs, which are perceived by distinct membrane-bound receptor complexes but stimulate similar signaling pathways in the infected or other proximal macrophages. In addition to the canonical signaling pathway through STAT1/STAT2/IRF9 (also known as the ISGF3) binding to IFN-stimulated response elements (ISREs) in gene promoters, leading to induction of a large number of IFN-stimulated genes (ISGs) and pro-inflammatory responses, both types of IFNs, in particular manifested using type I IFNs, also signal through STAT1 homodimers, which are more commonly associated with the IFNγ-mediated signaling pathway for classical activation (M1) macrophages. Other STAT heterodimers and homodimers (including STAT3-6) may also be activated but lead to production of anti-inflammatory and immunosuppressive IL-10 and IL-1Ra. Other STAT-independent signaling pathways including MAPK- and PI3K-pathways also may be activated, thereby exerting diverse effects in macrophages as well as other immune cells (such effects on T cell, in particular Treg cell ratio), which critically regulate the outcomes of virus-host interaction through, at least in part, the modulation of macrophage polarization [30,31].

After perception by the corresponding receptors, the canonical signaling pathway mediated by type I and type III IFNs leads to the activation and dimerization of STAT1 and STAT2, which further recruits IFN-regulatory factor (IRF)-9 to form an IFN-stimulated gene factor (ISGF)-3 complex. This complex translocates into the nucleus to promote the expression of a series of IFN-stimulated genes (ISGs) bearing different antiviral capacities (Figure 2) [30–31]. In addition to this canonical signaling pathway, recent studies have revealed that IFN-α/β are also effective at regulating other non-canonical signaling pathways mediated by other STAT homodimers (e.g., STAT1/STAT1, STAT3/STAT3, and so on to STAT6), cellular MAPK (mitogen-activated protein kinase) cascade, and PI3K/Akt/mTOR signaling [28–31,80,96]. IFN-α/β may signal through STAT1 homodimers, which are more commonly associated with the IFN-γ-mediated signaling pathway for M1 polarization [93,97], and other STAT homodimers, which are commonly associated with signaling pathways mediated by IL-6 (STAT3), IL-12 (STAT4), GM-CSF (STAT5), IL-4/IL-13 (STAT6), and IL-10 (STAT3 and STAT6), respectively [98]. These associations imply crosstalk between signaling pathways mediated by type I IFNs and other cytokines [96–99] and they indicate the multifunctional potency of type I IFNs in the regulation of cell differentiation and activation responses to these cytokines [28–31,98]. Because most cells have IFNAR receptors and are responsive to type I IFNs, differential expression of STAT isoforms and regulation of their dimerization may direct which pathway the IFN signaling influx is elicited [99]. For example, STAT1 and STAT2 are highly expressed in macrophages, and expression of STAT3 is more restricted to epithelial cells [96,99].

However, there is little data to show differential expression of STAT genes in macrophages at different activation statuses, particularly at the protein level. Using a RNA-Seq procedure, we have analyzed gene expression of all STAT genes in porcine alveolar macrophages repolarized at different activation statuses. We showed that all STAT genes (STAT1-4, 5a, 5b, and 6) are expressed in alveolar macrophages, with STAT1 and STAT2 having 10- to 200-fold higher expression levels than other STAT transcripts at 16 h post PRRSV-infection ([70], unpublished data). Therefore, it appears that STAT1- and STAT2-involved IFN-signaling pathways lead to M1-MaV status in this case [70,71]; however, it remains elusive in situations when, for example, type I IFNs induce M2 status via STAT3/STAT3 and STAT6/STAT6. It is likely that macrophages have a dynamic regulation of the relative ratio of STAT proteins corresponding to their tissue location and functional phenotypes, and that IFNs may play a dual role in M1- and M2-polarization as well as eminently for induction of the antiviral state [28–31,70,98]. In summary, as shown in Figure 2, diverse signaling pathways mediated by type I and type III IFNs have been discovered in different cell types [28–31] and may have potential to crosstalk with signaling pathways leading to phenotypes or either M1-like or M2-like polarization. Considering the canonical antiviral stimulation and all other signaling pathways leading to typical M1 and M2 characteristics, we propose that the antiviral state is an operative polarization status relatively independent of either M1 or M2 statuses [1,6,71]. Much is still unknown about the mechanisms that regulate type I IFNs either in canonical antiviral stimulation or switching to strengthen M1 or M2 statuses [28–30,98]; however, macrophage polarization progression mediated by the net result of these IFNs and crosstalk with other mediators is likely critical in determining the outcome of monocytotropic viral infections.

## Viruses Evolve to Adapt and Mediate Macrophage Polarization

In the above sections, we discussed some virus actions on macrophage polarization in the context of virus-host interaction. Because of the limited encoding capacity of viral genomes compared with the host, the evolution of viral mechanisms targeting macrophage polarization implies that overcoming the macrophage barrier (functionalized by polarization) is critical to viral infection [6,10,14]. Here we review this topic from the perspective of the virus. In general, monocytotropic viruses have evolved two mechanisms to avoid potent immune responses mediated by proper macrophage polarization. The first is to directly adapt to the existing favorable polarity of macrophage activation [100,101] and the second is to actively modulate the unfavorable status of macrophage polarization [86,87].

### Taking advantage of the macrophage pro-M2 status related to immature immunity in early life

Vulnerability to viral infections is much higher in neonates than adults. Fetal and neonatal immunity adapt to intrauterine survival and facilitates postnatal protection against extracellular pathogens; however, there is a window of susceptibility to intracellular pathogens such as viruses [100,101]. Corresponding to active tissue remodeling and angiogenesis activity, fetal and neonatal monocytic cells have a nonclassical monocyte phenotype with higher expression of scavenger receptors (CD36 and CD163), and Fc receptors (FcγRI and FcγRII), as well as cytokine/chemokine receptors CD115 (M-CSFR), CD116 (GM-CSFR), and CX3CR1, but lower expression of CCR2 and CCR5 than comparable adult cells. With or without PAMP stimulation, neonatal monocytic cells produce lower levels of Th1 cytokines, including IFN-γ and IL12 (supporting the clearance of intracellular pathogens), but higher levels of immunosuppressive cytokines such as IL-10 and TGF-β. However, PAMP stimulate fetal cells to produce adult concentrations of IL-1β, IL-6 and IL-23, which support Th17 cell differentiation and the clearance of extracellular pathogens [100,102]. Other factors including adenosine and other soluble factors in neonatal plasma appear to further skew this cytokine milieu, which inclusively nurtures monocytic cells toward a nonclassical M2-like status vulnerable to viral infection in early life [101].

Viral infections acquired early in life are often associated with a higher rate of viral replication, a greater risk of persistent (chronic) infection, and more severe disease compared with those acquired in later life. For example, children with perinatal HIV infections experience a rapid disease progression (several months compared with 10 years in adult patients), more severe clinical signs with more opportunistic infections, and little probability (<5% compared with 5–15% in adult patients) of becoming long-term non-progressors [101,102]. In most cases of RSV and rhinovirus infections, where clinical signs are lacking or mild in adults, neonates generally show severe allergic inflammation and an asthmatic syndrome. This virus-mediated asthmatic syndrome in neonatal animals is promulgated by IL-4-mediated M2 polarization of macrophages [24]. In addition, macrophages are present in all maternal-fetal compartments, including the placenta and endometrium, and successful pregnancy requires that the activation status of these utero-placental macrophages remains regulated throughout pregnancy [100]. It has been reported that utero-placental macrophages have a pro-M2 status to facilitate fetal development and manipulation of macrophage polarity by infectious agents can impact pregnancy outcomes [100]. To this end, PRRSV, causes severe respiratory infection in young pigs and infects pregnant sows in utero causing reproductive failure involving massive abortion storms, stillbirth, and mummified fetuses [10,71]. How PRRSV infection causes the imbalance of macrophage polarization in the maternal-fetal interface and in turn leads to reproduction failure, remains unknown. Clearly, some viruses are successful pathogens that have evolved to take advantage of the pro-M2 status to establish infections in some immunoprivileged sites (such as brain, placenta) or processes (such as fetal development) [100,101].

### Modulation of the progression of macrophage polarization

Multiple viral factors of monocytotropic viruses, which interfere with virus-host interactions, may potentially act to modulate the balance or progression of macrophage polarization. Because most of these viral mechanisms have been reviewed elsewhere [10,28–30,82,87,103,104], we will briefly discuss their prospective interaction with macrophage polarization.

- Virus-induced macrophage depletion: Because virus-permissive macrophages and other monocytic bystander cells serve as the first group of responders, induction of cell death in these cells provides a general strategy to subvert host defenses against the infections of monocytotropic viruses. Several viral proteins have proapoptotic activity. Prominent examples include the M protein of Dengue virus, influenza NS1, PB1-F2 protein and nucleoprotein (NP), and HIV Tat, gp120, Nef, and Vpu proteins. Direct induction of cell death in infected macrophages and bystander cells blocks acute antiviral responses, contributes to local tissue damage, and attenuates efficient progression of macrophage polarization toward M1- and MaV-statuses; all of these in turn contribute to compromised antiviral immunity, leading to high incidences of mortality or chronic viral persistence [14,79,103,104,105].
- Virus-mediated inflammatory and cytokine responses: As illustrated in Table 1 and discussed above, most monocytotropic viral infections dynamically alter inflammatory cytokine profiles during the infection process. For example, during high-pathogenic influenza infections massive production of pro-inflammatory cytokines (cytokine storm) is associated with the acute phase and severe immunopathology. In contrast, HIV is capable of switching infected macrophages to a M2-status through induction of IL-4 and IL-10 [12]. In either case, the deviation of the cytokine profiles leads to improper polarization of macrophages and is linked to inefficient antiviral immunity [14,75,81,82].
- Viral mechanisms targeting type I IFN production and signaling: As reviewed elsewhere, a plethora of viral proteins potently suppress or block the production and action of type I IFNs. Because of the multifunctional potency of type I IFNs in regulating signaling pathways leading to M1- and M2-polarization, the aberration of type I IFN production and action will potentially affect the progression of macrophage polarization. Given that the antiviral state is one polarity of macrophage activation, viral mechanisms targeting type I IFNs are among the most prominent factors affecting macrophage phenotypes and functionality [10,28–31].
- Virus-encoded IL-10 analogs (vIL-10): IL-10 is a pleiotropic cytokine with prominent immunosuppressive properties that polarizes macrophages to a M2c status. To date, vIL-10 analogs have been reported to be synthesized by multiple members of several DNA virus families. These viral genes may have evolved independently in each viral genome and obtained partial IL-10 molecular function to mimic cellular IL-10 activities to benefit the virus life cycle [86]. Some RNA viruses such as PRRSV, which generally have smaller genomes than those of typical DNA viruses, are alternatively capable of inducing cellular IL-10 production [10,83–86]. The presence of either vIL-10 or viral induction of cellular IL-10 facilitate pro-M2 polarization of macrophages and virus-mediated immunosuppression, which in turn benefits viral infection and persistence and dampens immune control of viral infection [83–86].
- Virus-encoded miRNA and other signaling pathways: Recently, some microRNA (miR) species have been identified in regulating macrophage activation status. For example, miR-223 and Let7a modulate inflammation and affect M2-polarization; in contrast, miR-511-3p attenuates M2-polarization [11,106]. Therefore, viruses may work through these host miRNA species or through encoding viral miRNA to influence macrophage polarization, thereby affecting the process of virus-host interaction [107]. Other signaling pathways potentially involved in viral regulation of macrophage polarization include sphingosine-1-phosphate (S1P) signaling pathway and PI3K/Akt/mTOR signaling pathway; however, exact mechanisms of regulation remain largely unknown [80–82]. In particular, the S1P signaling pathway has been implicated in regulation of cytokine storms in animals infected by pandemic influenza virus. This finding deserves further investigation to help design therapies that blunt cytokine storms and related virus-mediated immunopathology [81].

## Commensals and Endogenous Viral Factors May Educate Steady-State Macrophages Prior to Viral Infection

As discussed above in Section 3, the constitutive weak IFN-α/β signaling produced by monocytic cells is instructive in macrophage polarization and in mediating efficient antiviral immunity. Recently, the factors that mediate the constitutive production of low levels of type I IFNs have been identified. Abt et al. (2012) and Ganal et al. (2012) simultaneously reported that PAMP (including bacterial LPS and microbial nucleic acid) leaking from microbiota induces weak IFN tonic signaling and positions macrophages for efficient immune induction after virus infection. In contrast, germ-free animals without commensal microbiota lack this immune efficacy upon pathogenic infections [90–92].

Endogenous retroviruses (ERVs) are remnants of ancestral retroviral integration into the genome of germ-line cells constituting 4–10% of genome sequences in different animal species [108,109]. The expression of ERVs is closely scrutinized by cellular epigenetic factors at the DNA level and vigorously restricted by the immune system [110,111]. For example, mice that are deficient in producing mature T cells and antibodies exhibit high resurrection of ERVs in lungs and macrophages [111]. In addition, neonatal mice, with an immature immune system had higher expression of ERVs [112]. Our transcriptomic RNA-Seq data showed that ERV expression increased during macrophage M2-polarization but was suppressed at M1 and particularly a MaV status [70,88, unpublished data]. Therefore, whereas commensal bacterial PAMPs provide tonic signaling for instructive and efficient activation of macrophages [90–92], we propose that ERV expression in steady-state and M2-macrophages may serve as an intrinsic alarm that may contribute to the stochastic expression of type I IFNs and cytokines responsible for phenotypic diversity at a microscale of macrophage polarization [113].

## Concluding Remarks: Targeting Macrophage Polarization to Manage Virus-Host Interactions

For viral infections, particularly in monocytotropic cases, the paradigm of macrophage polarization provides a framework to integrate the antiviral state and to understand virus-host interactions with respect to virus pathogenic mechanisms and aberrant immune responses [1,6,14,71]. Through this framework, we suggest that prevention and treatment of viral diseases need not be focused solely on antiviral effectors against viruses, but may be managed to achieve immune/antimicrobial homeostasis (Figure 2). In this manner, many therapeutic designs against viral diseases may extend to regulating macrophage (and host) immune status rather than focusing principally on virus-killing [16,82,114–118]. As validated in mice, agents that increase M2a-differentation blunt RSV-mediated lung pathology [115] and protection from cytokine storms and lethality induced by pandemic influenza has been achieved by blocking TLR2 and TLR4 signaling or blocking endothelial S1P signaling [81]. Rotavirus infection was prevented and cured via the signaling pathway mediated by TLR5 and NOD-like receptor C4 (NLRC4), which led to production of IL-22 and IL-18 (mimicking the Th17-polarization) [118]. To this end, we and others have shown that modulation of lipid metabolism, such as suppression of acetyl CoA-carboxylase (ACC), manipulation of cholesterol metabolism, and epigenetic regulation [71,119,120], could re-polarize macrophages and significantly affect macrophage susceptibility to viral infections. The antiviral IFN system (i.e., the production and action of type I and type III IFNs) remains focused on control of viral infections. However, two recent studies have indicated that blockade of chronic type I IFN signaling facilitates restoration of effective immune status and ultimately leads to clearance of the persistent infection by lymphocytic choriomeningitis virus (LCMV) [121,122]. These findings emphasize the significance in studying viral infections and IFN-mediated antiviral responses within the paradigm of cell immune status and with a dynamic view of the virus-host interaction.

## Figures and Tables

**Table 1:** Monocytotropic viruses and pathogenic effect of macrophage manipulation/infection

Virus* (genome, family)	Macrophage-related primary infection cells/sites	Effect of manipulation/infection in monocytes, MΦs and DCs	Reference
**DENV** ((+)ssRNA, Flaviviridae)	Monocytes, MΦs and DCs in multiple tissues of IFN-αβγR KO mice	MΦ-depletion: Tenfold increase in systemic viral titer, and massive infiltration of monocytes	[32,33]
**RSV** ((-)ssRNA, Paramyxoviridae)	Blood monocytes, DCs, lung epithelial cells and MΦs in mice/humans	MΦ-depletion: Abolished local inflammatory cytokine peak at 1 dpi, and enhanced viral load in the lung at 4 dpi	[34,35]
**HIV1** ((+)ssRNA, Retroviridae)	Macrophages and T cells in humans	Deficiency of CCR5, a co-receptor that mediates HIV macrophage-tropism, showed resistance to HIV-1infection	[39,40]
**WNV** ((+)ssRNA, Flaviviridae)	Murine keratinocytes and skin-resident DCs, and probable peripheral MΦs and DCs mediating neuroinvasion	MΦ-depletion: Higher and extended viremia, and accelerated encephalitis and death. Inhibition of NOS activity of infiltrating MΦs relieved encephalitis and prolonged survival	[41–43]
**SARS-Cov** ((+)ssRNA, Coronaviridae)	Human respiratory epithelial cells, and antibody-enhanced infection of macrophages and immune cells	Depletion of alveolar MΦs 1–2 day before infection, (but not at 2 dpi), prevented lethal disease, and enhanced viral clearance	[44,45]
**IAV** (Segmented (-)RNA, Orthomyxoviridae)	Airway and lung epithelial cells, DCs, and MΦs of mice/humans/pigs	MΦ-depletion: Strain-dependent exacerbation of viral replication, increased airway inflammation and viral pneumonia	[36–38]
**CSFV** ((+)ssRNA, Flaviviridae)	Porcine blood monocytes/macrophages	Viral infection stimulated arginase-1 (ARG-1) but suppressed nitric oxide synthase (iNOS) expression, i.e., induced M1-M2 repolarization	[50,51]
**PrV** (dsRNA, Hepesviridae)	Porcine lung epithelial cells and MΦs and spread via infected blood monocytes	Acute IFN-α response is important in diminishing the spread of PrV in the connective tissue but not in epithelial cells (IFN cell preferences)	[52–54]
**ASFV** (dsRNA, Asfarviridae)	Primarily and persistently infected tissuemonocytes/MΦs and fibroblasts in multiple tissues	Massive M1 polarization served as a modulator of the viral pathogenesis including pulmonary edema, hemorrhage, and lymphoid depletion that characterize the disease	[55,56]
**PCV2** (ssDNA, Cirvoviridae)	Monocyte/MΦ lineage cells, including alveolar MΦs, are the major target cells	Acute infection reduced alveolar MΦs phagocytosis and microbicidal capability; and persistence increased inflammatory and pro-apoptotic responses, which led to lymphopenia and immunosuppression	[57,58]
**FMDV** ((+)ssRNA, picornaviridae)	Early infection of porcine T and B cells caused viremia; immunocomplex promoted productive infection and killing of mDCs	Increase IL-10 production in infected DCs, loss of pDC cell function coincides with lymphopenia in FMDV-infected pigs; macrophage depletion in vaccinated mice severely decreased vaccine protection	[59–63]
**PRRSV** ((+)ssRNA, Arteriviridae)	Tissue macrophages, monocytes and mDCs especially those in reproductive and respiratory tracts.	Massive cell death of infected monocytic cells; increase of IL-10 and reduction of phagocytic, microbicidal, pro-inflammatory, and antigen-presentation activity in MΦs and DCs. Pathogenicity-related suppression of IFN-α production in pDCs	[64–66]

* ASFV: African Swine Fever Virus; CSFV: Classical Swine Fever Virus; DENV: Dengue Virus; FMDV: Foot and Mouth Disease Virus; HIV1: Human Immunodeficiency Virus 1; IAV: Influenza A Virus; PCV2: Porcine Circovirus-2; PRRSV: Porcine Reproductive and Respiratory Syndrome Virus; PrV: Porcine Pseudorabies Virus; RSV: Respiratory Syncytial Virus; SARS-Cov: Severe Acute Respiratory Syndrome Coronavirus; WNV: West Nile Virus
